# Supplementary material for: Targeted V1 comodulation supports task-adaptive sensory decisions
Source: Nat Commun. 2023 Nov 30;14:7879. doi: 10.1038/s41467-023-43432-7 (PMC10689451; doi:10.1038/s41467-023-43432-7)
Supplement: Supplementary file 3 — Reporting Summary [file 41467_2023_43432_MOESM3_ESM.pdf]

nature portfolio

Corresponding author(s): Caroline Haimerl

Last updated by author(s): Apr 28, 2023

Reporting Summary

Nature Portfolio wishes to improve the reproducibility of the work that we publish. This form provides structure for consistency and transparency in reporting. For further information on Nature Portfolio policies, see our Editorial Policies and the Editorial Policy Checklist.

Statistics

For all statistical analyses, confirm that the following items are present in the figure legend, table legend, main text, or Methods section.

n/a	Confirmed	
☐	☒	The exact sample size (*n*) for each experimental group/condition, given as a discrete number and unit of measurement
☐	☒	A statement on whether measurements were taken from distinct samples or whether the same sample was measured repeatedly
☐	☒	The statistical test(s) used AND whether they are one- or two-sided<br>*Only common tests should be described solely by name; describe more complex techniques in the Methods section.*
☐	☒	A description of all covariates tested
☒	☐	A description of any assumptions or corrections, such as tests of normality and adjustment for multiple comparisons
☐	☒	A full description of the statistical parameters including central tendency (e.g. means) or other basic estimates (e.g. regression coefficient) AND variation (e.g. standard deviation) or associated estimates of uncertainty (e.g. confidence intervals)
☐	☒	For null hypothesis testing, the test statistic (e.g. *F*, *t*, *r*) with confidence intervals, effect sizes, degrees of freedom and *P* value noted<br>*Give P values as exact values whenever suitable.*
☒	☐	For Bayesian analysis, information on the choice of priors and Markov chain Monte Carlo settings
☒	☐	For hierarchical and complex designs, identification of the appropriate level for tests and full reporting of outcomes
☐	☒	Estimates of effect sizes (e.g. Cohen's *d*, Pearson's *r*), indicating how they were calculated

Our web collection on statistics for biologists contains articles on many of the points above.

Software and code

Policy information about availability of computer code

Data collection	V1: 10 x 10 microelectrode array (Blackrock Microsystems)<br>MT units: single electrode (Fred Haer Corporation) or 24-channel probes (either Alpha Omega Linear Micro Arrays or Plexon V-Probes
Data analysis	all analysis was done in python, the code is provided on github (https://github.com/CarolineHaimerl27/modulator_guided_decoding.git). The following packes were used: numpy, matplotlib, sklearn, pandas, scipy, pickle, pytorch (see Code availability statement in manuscript for details and citations).

For manuscripts utilizing custom algorithms or software that are central to the research but not yet described in published literature, software must be made available to editors and reviewers. We strongly encourage code deposition in a community repository (e.g. GitHub). See the Nature Portfolio guidelines for submitting code & software for further information.

Data

Policy information about availability of data

All manuscripts must include a data availability statement. This statement should provide the following information, where applicable:

- Accession codes, unique identifiers, or web links for publicly available datasets
- A description of any restrictions on data availability
- For clinical datasets or third party data, please ensure that the statement adheres to our policy

A 10 by 10 microelectrode array (Blackrock Microsystems) was implanted in area V1 and a recording chamber was used for area MT. For details on experiments

please see Ruff and Cohen (2016) 10.1523/JNEUROSCI.0610-16.2016 where the data was previously published.
The data has previously been published in Ruff and Cohen (2016). It is available upon reasonable request. The python code for the theory and analysis together with an example dataset is provided on github (https://github.com/CarolineHaimerl27/modulator_guided_decoding.git).

Research involving human participants, their data, or biological material

Policy information about studies with human participants or human data. See also policy information about sex, gender (identity/presentation), and sexual orientation and race, ethnicity and racism.

Reporting on sex and gender	N/A
Reporting on race, ethnicity, or other socially relevant groupings	N/A
Population characteristics	N/A
Recruitment	N/A
Ethics oversight	N/A

Note that full information on the approval of the study protocol must also be provided in the manuscript.

Field-specific reporting

Please select the one below that is the best fit for your research. If you are not sure, read the appropriate sections before making your selection.

☒ Life sciences ☐ Behavioural & social sciences ☐ Ecological, evolutionary & environmental sciences

For a reference copy of the document with all sections, see nature.com/documents/nr-reporting-summary-flat.pdf

Life sciences study design

All studies must disclose on these points even when the disclosure is negative.

Sample size	67 blocks of 20 recordings sessions across two monkeys were analyzed for the relevant task condition and 20 blocks for the control condition. A block had on average 54 trials that resulted in either a miss or a hit and were analyzed. On average there were 88 units in a population of a data block (minimum 52 and maximum 95). For details see methods/supplement. We have validated that ground truth recovery on artificial data with similar statistics is possible.
Data exclusions	Neural units are excluded if the response to either one of the stimuli does not change by at least 10% compared to their baseline value as those units cannot be differentiated from noise. Units with very low rate (<0.1Hz) or a very high Fano Factor (>5 sd above population average) are also excluded. We excluded blocks that had less than 20 trials that ended in a miss or hit behaviorally. For details see methods/supplement.
Replication	replication was done across animals and across units collected during different experimental sessions
Randomization	The type of experimental task blocks were randomly interleaved in a session. No other randomization was done. Randomization was not required because there were no treatment groups that received different experimental conditions.
Blinding	Blinding was not required because there were no treatment groups that received different experimental conditions.

Reporting for specific materials, systems and methods

We require information from authors about some types of materials, experimental systems and methods used in many studies. Here, indicate whether each material, system or method listed is relevant to your study. If you are not sure if a list item applies to your research, read the appropriate section before selecting a response.

Materials & experimental systems

n/a	Involved in the study
☒	☐ Antibodies
☒	☐ Eukaryotic cell lines
☒	☐ Palaeontology and archaeology
☐	☒ Animals and other organisms
☒	☐ Clinical data
☒	☐ Dual use research of concern
☒	☐ Plants

Methods

n/a	Involved in the study
☒	☐ ChIP-seq
☒	☐ Flow cytometry
☒	☐ MRI-based neuroimaging

Animals and other research organisms

Policy information about studies involving animals; ARRIVE guidelines recommended for reporting animal research, and Sex and Gender in Research

Laboratory animals	adult male rhesus monkeys (macaca mulatta, 8 and 9 kg, 6 and8 years old)
Wild animals	No wild animals were used in the study.
Reporting on sex	both animals male
Field-collected samples	No field-collected sampled were used in the study.
Ethics oversight	Institutional Animal Care and Use Committees of the University of Pittsburgh and Carnegie Mellon University

Note that full information on the approval of the study protocol must also be provided in the manuscript.
